# Honey bee hives decrease wild bee abundance, species richness, and fruit count on farms regardless of wildflower strips

**DOI:** 10.1038/s41598-021-81967-1

**Published:** 2021-02-05

**Authors:** G. M. Angelella, C. T. McCullough, M. E. O’Rourke

**Affiliations:** 1 School of Plant and Environmental Sciences, Virginia Tech, Blacksburg, VA USA; 2 Present Address: USDA, Agricultural Research Service, Temperate Tree Fruit and Vegetable Research Unit, 5230 Konnowac Pass Road, Wapato, WA 98951 USA; 3 Present Address: Department of Entomology, Virginia Tech, Blacksburg, VA USA; 4 Present Address: USDA, National Institute of Food and Agriculture, Kansas City, MO USA

**Keywords:** Agroecology, Entomology

## Abstract

Pollinator refuges such as wildflower strips are planted on farms with the goals of mitigating wild pollinator declines and promoting crop pollination services. It is unclear, however, whether or how these goals are impacted by managed honey bee (*Apis mellifera* L.) hives on farms. We examined how wildflower strips and honey bee hives and/or their interaction influence wild bee communities and the fruit count of two pollinator-dependent crops across 21 farms in the Mid-Atlantic U.S. Although wild bee species richness increased with bloom density within wildflower strips, populations did not differ significantly between farms with and without them whereas fruit counts in both crops increased on farms with wildflower strips during one of 2 years. By contrast, wild bee abundance decreased by 48%, species richness by 20%, and strawberry fruit count by 18% across all farm with honey bee hives regardless of wildflower strip presence, and winter squash fruit count was consistently lower on farms with wildflower strips with hives as well. This work demonstrates that honey bee hives could detrimentally affect fruit count and wild bee populations on farms, and that benefits conferred by wildflower strips might not offset these negative impacts. Keeping honey bee hives on farms with wildflower strips could reduce conservation and pollination services.

## Introduction

Declining pollinator populations coupled with increasing pollinator-dependent crop production worldwide makes it crucial to understand how management techniques can enhance on-farm pollinator conservation and crop pollination ecosystem services^1–3^. Research in native plant systems suggests competition between managed and wild bees over floral resources can suppress wild bee abundance and plant seed set^4–7^. This may be especially true under resource limitations^6–8^, which can increase niche overlap between honey bees and wild pollinators and heighten negative impacts on wild pollinator populations^9–12^. Competition from managed bees can affect wild pollinator populations and crop production in agroecosystems as well^13–17^. However, we do not yet fully understand the factors shaping honey bee–wild bee interactivity on farms, including whether or how modifying floral resource availability modulates outcomes. Understanding how floral resource supplementation influences the effects of bee competition is necessary to maximize ecosystem services in agricultural production.

Pollinator refuge habitats (often referred to as wildflower strips) are designed to supplement floral resources and mitigate pollinator population and pollination service declines. Planting these habitats on farms is federally subsidized in some countries through programs such as Agri-Environment Schemes in Europe^18^, and the Natural Resources Conservation Service’s (NRCS) Environmental Quality Incentives Program and the Farm Service Agency’s Conservation Reserve Program in the USA^19,20^. Wildflower strips are designed to provide blooms throughout the growing season (e.g., FSA^21^) to enhance local populations of wild and managed pollinators by aggregating them on farms and supporting greater population growth^22^. They can increase both native bee^23–25^ and managed honey bee^26^ populations as well as crop yields^27–30^, but no studies to date have examined the impacts maintaining honey bee hives on farms with wildflower strips has on these beneficial services.

There is well-documented evidence that managed bees, particularly honey bees (*Apis mellifera* L.), can have negative effects on wild pollinators within natural systems^4–7^. Competition can also reduce seed set in native plants when honey bees drive down floral visitations by wild pollinators but cannot pollinate as effectively as their wild counterparts^31–33^. Since evidence suggests many pollinator-dependent crops respond more strongly to pollination services from wild pollinators than honey bees^34^, competition could conceivably decrease yield in many crop systems as well. However, the outcome of honey bee and wild pollinator co-occurrence in crops on yield seems to be context dependent: studies report effects on yield ranging from negative^14,16,17^, to positive or even synergistic^13,15,35^. This inconsistency illustrates the need to identify how underlying factors shape the outcome of wild bee–honey bee interactions. Moreover, it is unknown whether additional floral resources provisioned by wildflower strips could alleviate resource limitations and competition among pollinators.

**Figure 1:**
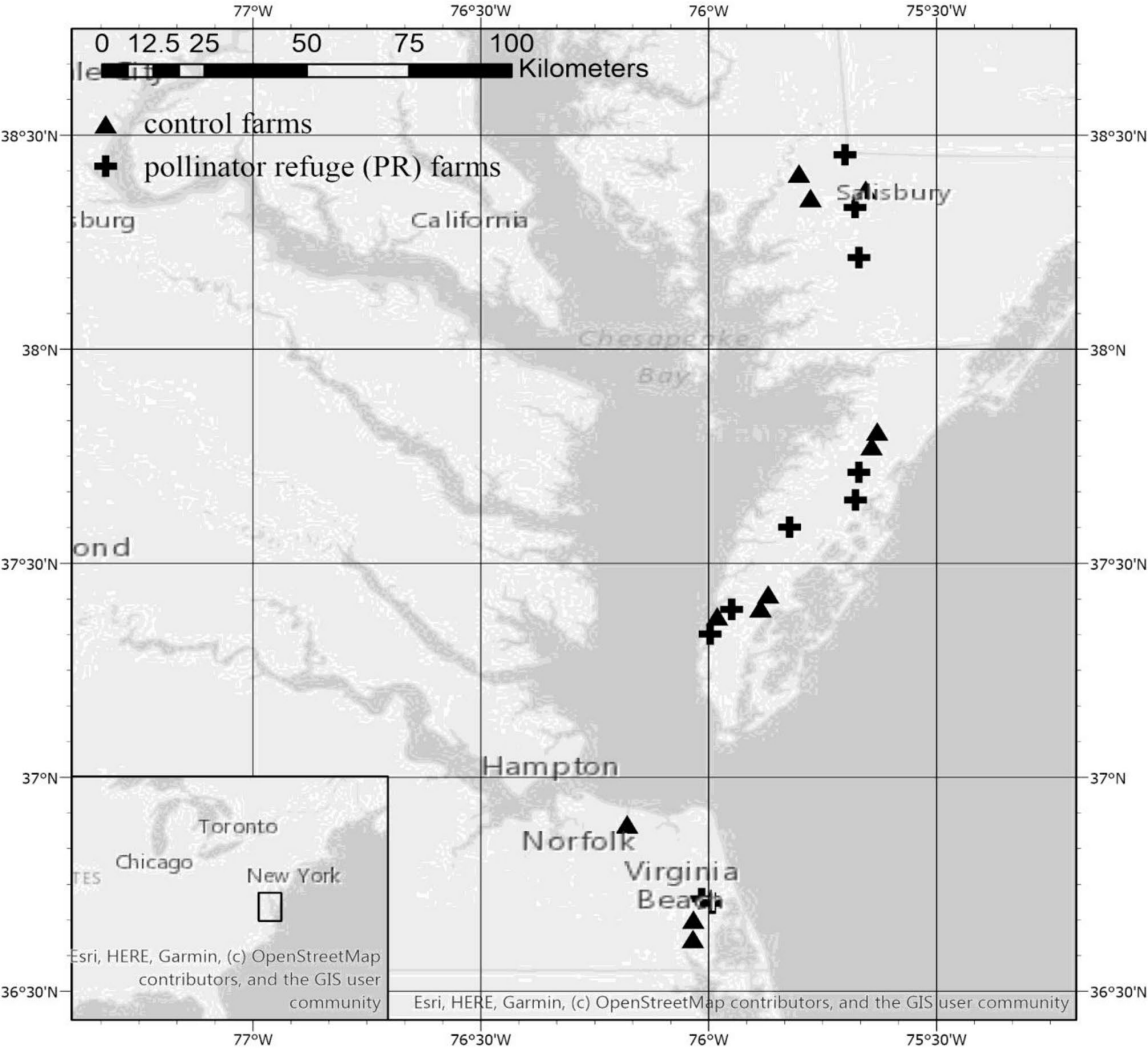
Map of farm locations. Farms planted with wildflower strips (pollinator refuge farms) and decimal degree longitudinal and latitudinal coordinates indicated. (See Table S1 for more details).

This study examines wild bee (i.e., non-*Apis* spp.) conservation and crop pollination value relative to wildflower strips, honey bee hives, and their interactions. Specifically, we wanted to know how wildflower strips and honey bee hives would independently influence wild bee abundance and fruit production on farms, and whether hives would enhance or detract from wildflower strip-mediated services. To answer these questions, we surveyed wild bee communities and measured strawberry and winter squash fruit count on a network of farms with or without wildflower strips and honey bee hives (Fig. 1).

## Results

The relative abundance and species diversity of blooms in field margins vs. wildflower strips varied by season. In early spring, bloom density was greater in field margins (μ = 85.30 ± 45.20) than in the wildflower strips (μ = 3.56 ± 1.54) (*Z* = − 3.29, *P* = 0.0010) but did not differ in diversity (field margins μ = 1.58 ± 0.16, wf strips μ = 1.30 ± 0.18) (*P* = 0.28). Contrarily, in mid-summer there was no difference in bloom density between field margins (μ = 34.18 ± 10.74) and wildflower strips (μ = 17.26 ± 9.51) (*P* = 0.52), but diversity was much greater in wildflower strips (field margins μ = 1.36 ± 0.36, wf strips μ = 2.88 ± 0.36) (*F* = 9.14, *P* = 0.0073).

### Bees

**Figure 2:**
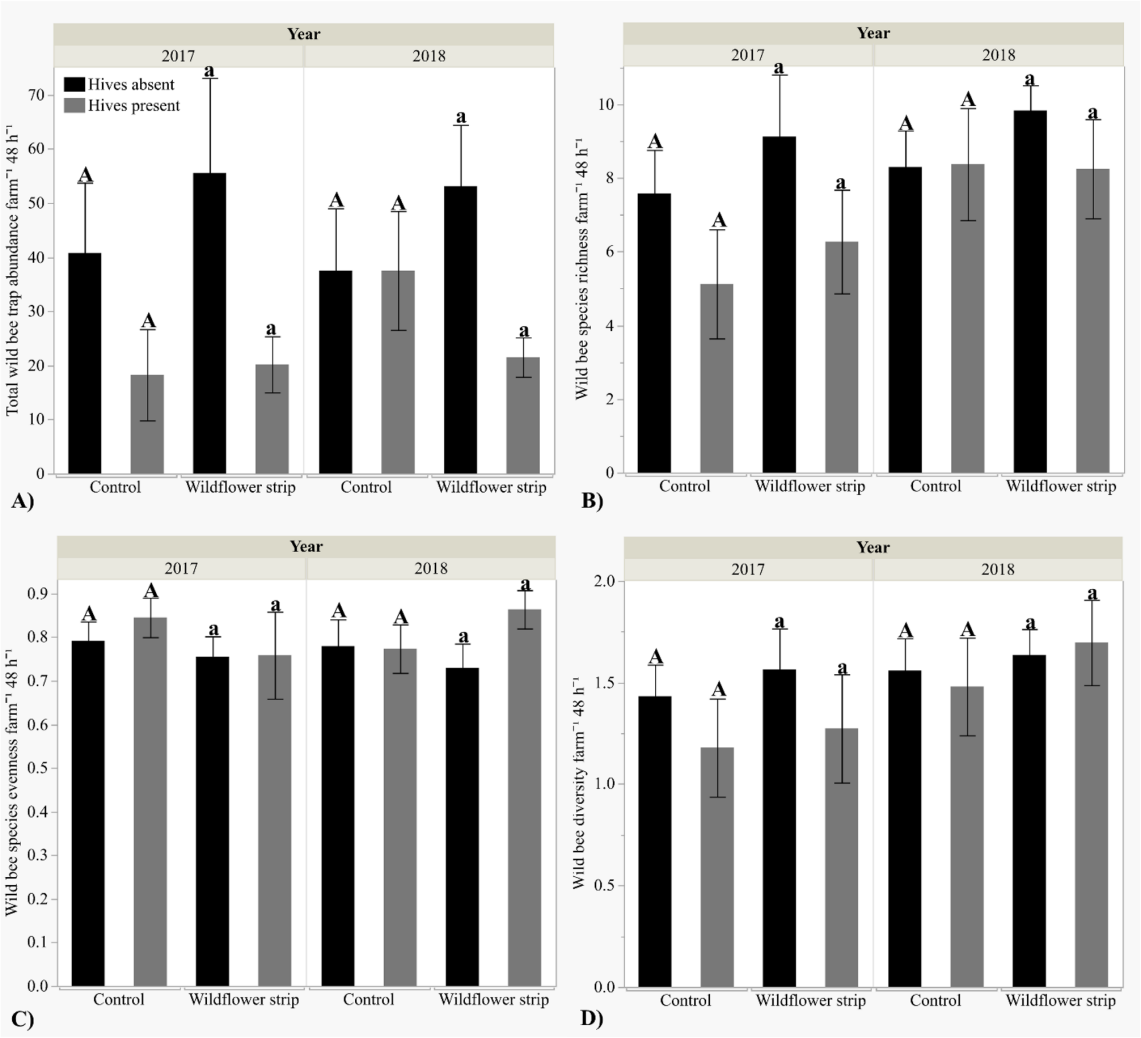
Mean total wild bee abundance, species richness, Shannon–Wiener diversity index, and species evenness (± SE) per farm by wildflower strip and honey bee hive presence/absence. Hive treatment means are compared within each wildflower strip treatment: the same letter indicates means are not statistically significant (α = 0.05). (**A**) Abundance, (**B**) Species richness, (**C**) Diversity, (**D**) Evenness.

**Figure 3:**
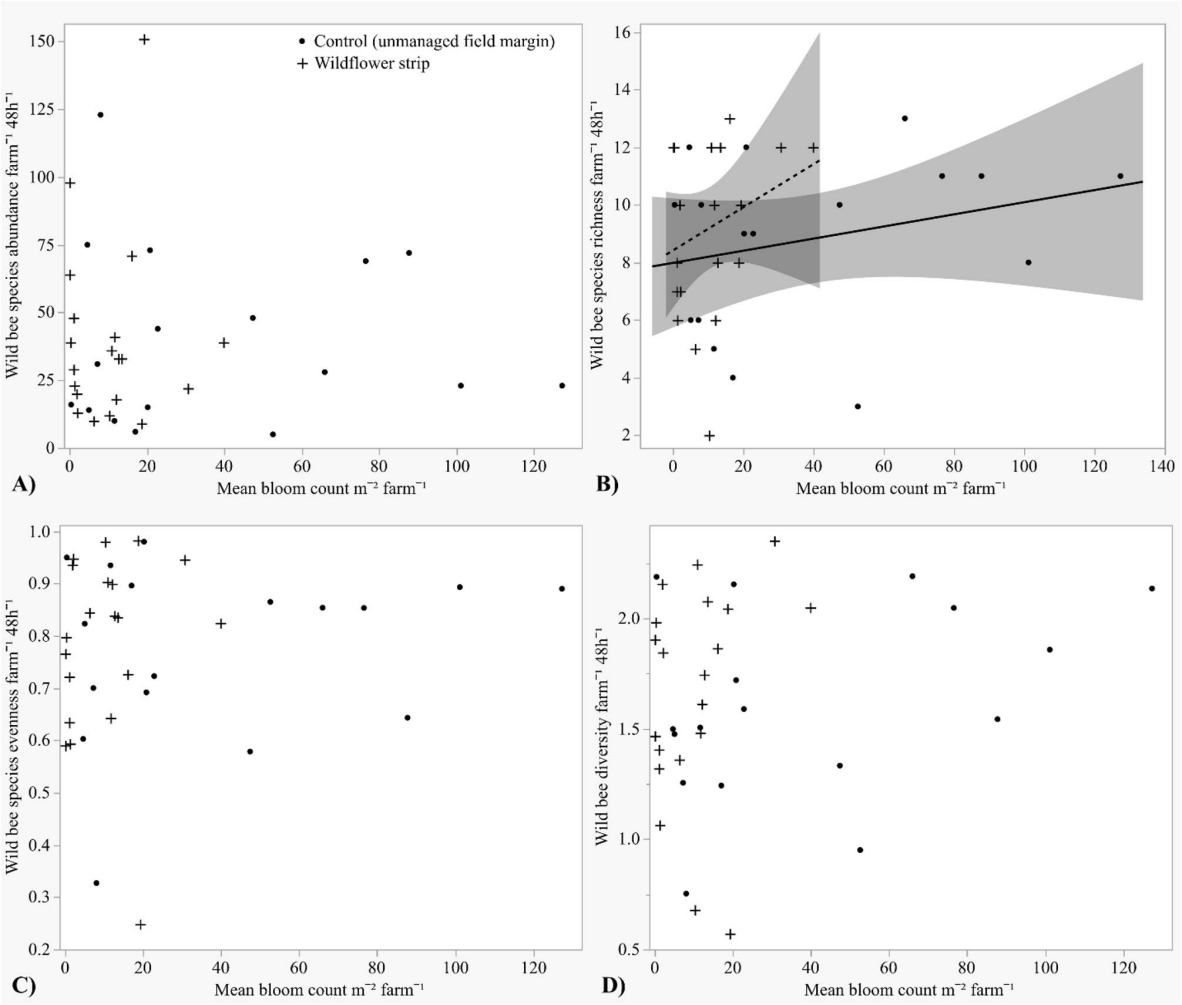
Mean total wild bee abundance, species richness, Shannon–Wiener diversity index, and species evenness (± SE) per farm by bloom density (mean bloom counts m^−2^) within wildflower strips or within unmanaged field margins on control farms. (**A**) Abundance, (**B**) Species richness, (**C**) Evenness, (**D**) Diversity.

Wildflower strips did not significantly increase wild bee abundance [+ wf strips μ = 37.82 ± 5.73, − wf strips μ = 34.47 ± 5.82], species richness (+ wf strips µ = 8.36 ± 0.65, − wf strips µ = 7.42 ± 0.64), evenness (+ wf strips µ = 0.77 ± 0.033, − wf strips µ = 0.80 ± 0.026), or Shannon–Wiener diversity (+ wf strips µ = 1.53 ± 0.10, − wf strips µ = 1.42 ± 0.095) (Fig. 2A–D, Table 1). Wild bee diversity was significantly greater in mid-summer (µ = 1.67 ± 0.074) than early spring (µ = 1.29 ± 0.11) but did not differ by year (Table 1). Neither wild bee abundance, species richness, nor evenness differed by season or year (Table 1). Although wild bee species richness did not significantly differ by wildflower strip presence/absence on farms, it increased with wildflower strip bloom density (*Z* = 2.21, *P* = 0.027) but not bloom density in unmanaged field margins on control farms (*P* = 0.31) (Fig. 3B). However, bloom density did not affect wild bee abundance (field margins *P* = 0.63, wf strips *P* = 0.31), evenness (field margins *P* = 0.42, wf strips *P* = 0.94), or diversity (field margins *P* = 0.16, wf strips *P* = 0.21) (Fig. 3A,C,D), and bloom species diversity did not affect any wild bee metric: abundance (field margins *P* = 0.63, wf strips *P* = 0.31), species richness (field margins *P* = 0.60, wf strips *P* = 0.87), diversity (field margins *P* = 0.47, wf strips *P* = 0.87), or evenness (field margins *P* = 0.58, wf strips *P* = 0.57).

**Table 1:** Wild bee survey data (abundance, species richness, Shannon–Wiener diversity, and species evenness) and fruit count data in response to wildflower strip (WF) and hive presence/absence, year, and/or season.

Response	WF	Hive	Year	WF*Year	Hive*Year	WF*Hive	WF*Hive*Year	Season
Wild bee abundance	*P* = 0.42	***Z = − 2.41 P = 0.016***	*P* = 0.86	–^α^	*P* = 0.087	–^α^	–^α^	*P* = 0.13
Wild bee richness	*P* = 0.50	***Z = − 2.03, P = 0.042***	*P* = 0.50	–^α^	*P* = 0.098	–^α^	–^α^	***Z = 2.45, P = 0.014***
Wild bee diversity	*P* = 0.42	*P* = 0.45	*P* = 0.085	–^α^	–^α^	–^α^	–^α^	***Z = 3.10, P = 0.0031***
Wild bee evenness	*P* = 0.81	*P* = 0.65	*P* = 0.88	–^α^	–^α^	–^α^	–^α^	***Z = 2.08, P = 0.043***
Strawberry fruit count	***Z = 2.10, P = 0.036***	***Z = 2.43, P = 0.015***	***Z = 5.77, P < 0.001***	***Z = − 2.02, P = 0.043***	–^α^	–^α^	–^α^	
Winter squash fruit count	*P* = 0.60	*P* = 0.34	***Z = − 3.15, P = 0.0017***	–^α^	***Z = − 2.60, P = 0.0093***	–^*β*^	–^*β*^	

Significant predictors (*P* < 0.05) are bolded.

^α^Nonsignificant interactions (α = 0.1) dropped from analyses.

^*β*^Interaction not included in analysis due to limited winter squash fruit count in 2018.

Honey bee hive presence was associated with a 48% decrease in wild bee abundance (+ hives μ = 24.00 ± 3.71, − hives μ = 46.31 ± 6.40), and a 20% decrease in species richness (+ hives μ = 6.94 ± 0.73, − hives μ = 8.69 ± 0.55) (Fig. 2A–B), whereas diversity (+ hives µ = 1.40 ± 0.12, − hives µ = 1.55 ± 0.077) and evenness (+ hives µ = 0.81 ± 0.034, − hives µ = 0.76 ± 0.026) did not differ significantly (Table 1). The honey bee hive effects on both wild bee abundance and species richness were marginally affected by year (Table 1), with overall differences of slightly greater magnitude occurring in 2017 than 2018 (Fig. 2A,B). Total honey bee abundance within traps was low but nevertheless greater on farms with hives than without (+ hives μ = 1.20 ± 0.57, − hives μ = 0.095 ± 0.057) (*Z* = 2.19, *P* = 0.029), and did not vary by year (*P* = 0.12) or season (*P* = 1.00).

### Fruit count

**Figure 4:**
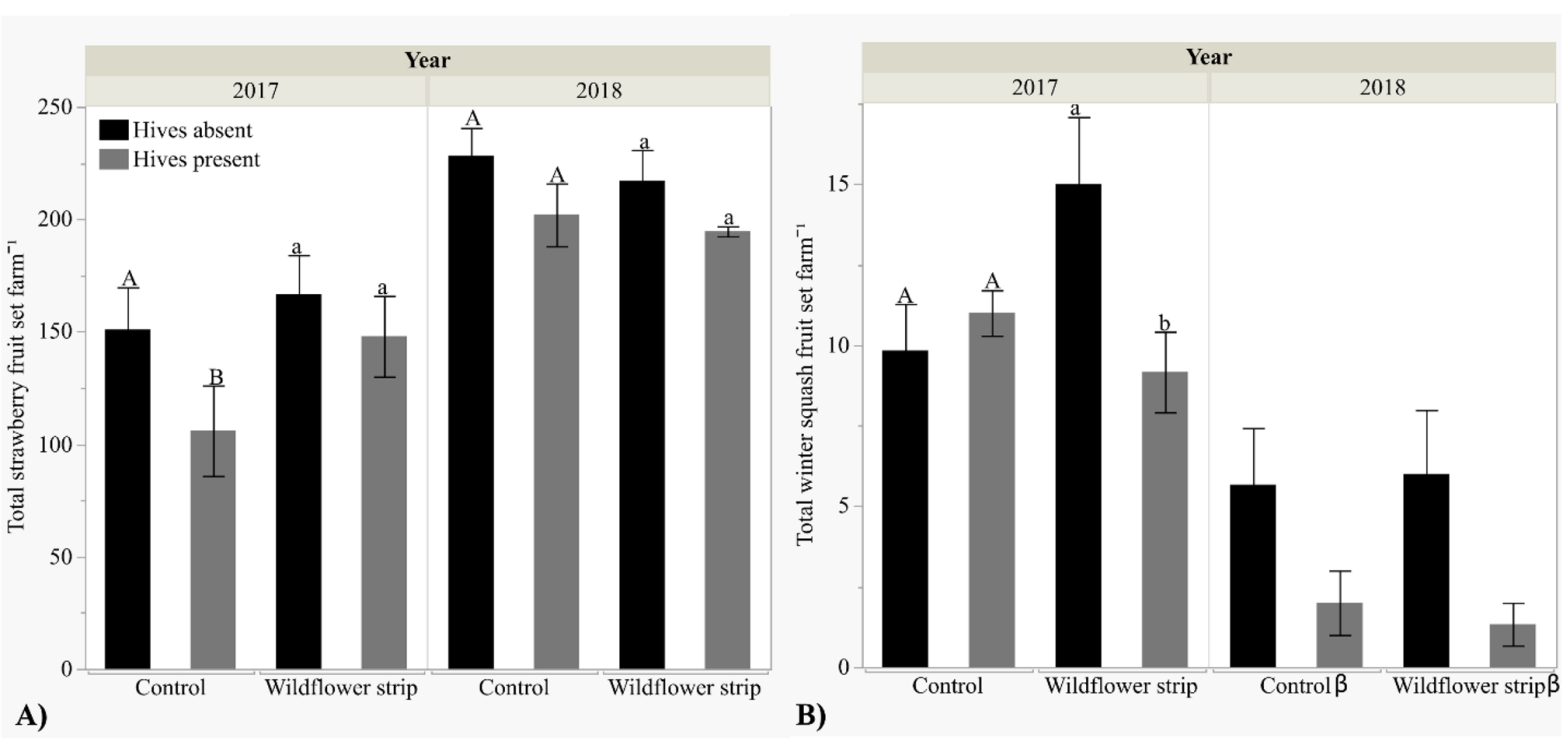
Mean total fruit count (± SE) per farm by wildflower strip and honey bee hive presence/absence on farms. Hive treatment means are compared within each wildflower strip treatment: the same letter indicates means are not statistically significant (α = 0.05), and β indicates sample sizes are insufficient for means comparisons. (See Supplemental Table S4 for sample size details.) (**A**) Strawberries, (**B**) Winter squash.

Wildflower strips significantly increased overall strawberry and not winter squash fruit count (Table 1). However, upon closer inspection, wildflower strips enhanced fruit counts of both during 2017 (strawberry: + wf strips µ = 157.30 ± 12.22, − wf strips µ = 124.50 ± 14.97; *t* = − 2.51, *P* = 0.018) (squash: + wf strips µ = 11.11 ± 1.40, − wf strips µ = 10.09 ± 0.84; *Z* = 2.33, *P* = 0.020) but not 2018 (strawberry: + wf strips µ = 209.67 ± 9.60, − wf strips µ = 215.10 ± 9.85; *P* = 0.55) (squash: + wf strips µ = 5.71 ± 1.64, − wf strips µ = 5.43 ± 1.46; *P* = 0.82) (Fig. 4A,B, Table 1). Total strawberry fruit count per farm was greater in 2018 than 2017 (2017 µ = 140.90 ± 10.13, 2018 µ = 212.53 ± 6.73) but the reverse was true for winter squash (2017 µ = 10.55 ± 0.77, 2018 µ = 5.57 ± 1.06) (Table 1).

Strawberry fruit count decreased by 18% across all farms with hives (+ hives µ = 156.89 ± 12.62, − hives µ = 192.00 ± 10.22) while the effect of hives on winter squash fruit count was less consistent (Fig. 4A,B, Table 1). Winter squash fruit count decreases across all farms with hives were not significant in 2017 (2017 + hives µ = 9.90 ± 0.82, − hives µ = 11.20 ± 1.31; *P* = 0.34), but they were in 2018 (2018 + hives µ = 2.00 ± 0.41, − hives µ = 7.00 ± 1.20; *t* = 3.16 *P* = 0.0038) (Table 1). Separately analyzing 2017 data with a hive by wildflower strip interaction showed fruit count differed by hive presence on farms with wildflower strips (+ hives µ = 9.17 ± 1.25, − hives µ = 15.00 ± 2.08; *Z* = 2.45, *P* = 0.014) but not without wildflower strips (+ hives µ = 11.00 ± 0.71, − hives µ = 9.57; *P* = 0.57) (hive*wildflower strip interaction: *Z* = − 2.14, *P* = 0.033) (Fig. 4B). Thus, winter squash fruit count decreased on farms with wildflower strips both years but only during 2018 on control farms. We were unable to analyze hive by wildflower strip interactions in the winter squash model with both years combined because of insufficient sample size in 2018 (Supplemental Table S4).

**Figure 5:**
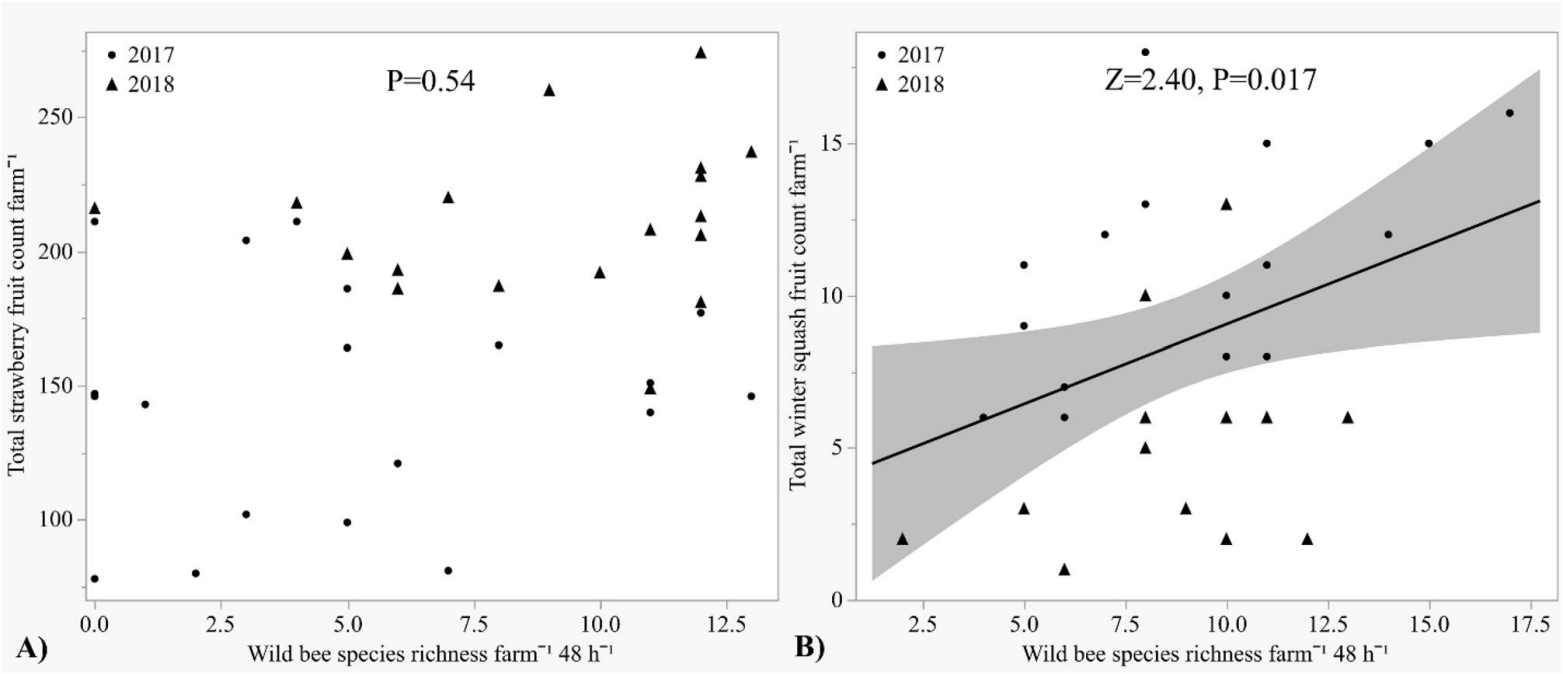
Total strawberry and winter squash fruit count analyzed by wild bee species richness: ▲ = 2017, ● = 2018. (**A**) Strawberry fruit count, (**B**) Winter squash fruit count.

Winter squash fruit count increased with greater wild bee species richness (*Z* = 2.40, *P* = 0.017), but strawberry fruit count did not (*P* = 0.54) (Fig. 5A,B). Fruit count was unaffected by wild bee abundance (*P* = 0.52, *P* = 0.27), diversity (*P* = 0.91, *P* = 0.29), or evenness (*P* = 0.13, *P* = 0.23) in both strawberry and winter squash, respectively. Neither honey bee abundance in traps (strawberries: *P* = 0.58; winter squash: *P* = 0.58), hive distance (strawberries: *P* = 0.97; winter squash: *P* = 0.52), nor the number of hives per farm (strawberries: *P* = 0.68; winter squash: *P* = 0.40) predicted changes in fruit count. The effect of wild bee abundance, species richness, diversity, evenness, honey bee abundance, number of hives, or hive distance on fruit count did not vary by year.

## Discussion

Wildflower strips induced responses ranging from neutral to limitedly positive in wild bee populations and fruit count, whereas responses to honey bee hives on farms were largely negative. There were no measurable changes in wild bee populations on farms with wildflower strips, although wild bee species richness increased with greater wildflower strip bloom density, but wildflower strips enhanced fruit count in one of 2 years. Meanwhile, there were marked overall decreases in wild bee abundance (48%), species richness (20%), and strawberry fruit count (18%) on farms with hives, regardless of wildflower strip presence or absence. Winter squash fruit count also decreased on farms with hives, but the effect was only consistent on farms with wildflower strips. Thus, negative effects associated with honey bee hive presence were as strong or stronger on farms with wildflower strips than on those without.

The lack of variation we observed in wild bee populations relative to supplementing farms with wildflower strips differs from previous work^23–25^. Nevertheless, the positive relationship between wild bee species richness and wildflower strip bloom density suggests significant treatment effects could arise at greater bloom densities. We observed particularly low bloom densities in the wildflower strips relative to field margins during the early spring; extending the bloom coverage of wildflower strips by including species with earlier phenologies could improve floral resource availability and attraction to wild bee species active earlier in the year. However, greater bloom densities within wildflower strips in mid-summer did not manifest in seasonal variation in wild bee populations on farms with wildflower strips, suggesting greater bloom densities in the summer may be needed to enhance wild bee populations as well. An alternative explanation for the lack of wildflower strip effect could be effects from the surrounding landscape. While we did not incorporate effects of surrounding landscape into analyses, its composition can modify the strength of wildflower strip effects as well by supporting nearby wild pollinator populations or even competing with wildflower strips in drawing pollinator activity^36–38^. For example, areas with relatively robust natural habitat, such as the Eastern Shore of the Mid-Atlantic, U.S., may demonstrate less of an effect from wildflower strips than areas with an intermediate proportion of natural habitat in the landscapes surrounding farms^39,40^. Additionally, while necessary in our study due to logistical constraints, pan and blue vane traps often under-sample bees relative to other methods such as netting at flowers or floral visitation observations^41,42^. For example, in the U.K., pan traps collected a lower proportion of pollinator populations where floral resources were abundant than where they were scarce due to competing sources of attractive stimuli^43^. Finally, shared floral resources can facilitate pathogen or parasite spillover^44,45^, which could counteract the benefits of supplemental floral resources on farms. It would be of interest to determine how wildflower strips alter pathogen and parasite transmission dynamics and pollinator population health on farms.

The changes in wild bee abundance, species richness, and evenness we observed on farms with hives could suggest competition of some kind is occurring between wild bees and honey bees. This would support previously documented observations of honey bee competition leading to declines in wild pollinator abundance^46–49^ and/or richness^4,50,51^. Exploitative competition, in which honey bees out-forage wild species and deplete shared floral resources, is thought by some to be the primary driver of negative outcomes in wild pollinator populations^46,49,52^. And yet, supplemental floral resources provided by wildflower strips did not alleviate the negative effect of honey bee hive presence on wild bees in our study, as would be predicted under limited floral resources. However, wild bee populations did not measurably benefit from wildflower strips on farms and we cannot determine the extent to which they were utilized relative to control farm field margins, as trap counts cannot infer foraging behavior^41,42^. An additional possible mechanism is that honey bee foraging within an area can reduce wild bee floral visitation^51^; thus, actively foraging honey bees on farms could have depressed wild bee activity. Behavioral studies incorporating floral visitation observations are needed to determine how and why wild bee populations vary between farms with and without honey bee hives. Moreover, pathogen or parasite spillover from managed bees can impact wild bee populations as well^44^. Future studies elucidating the behavioral and/or pathological mechanisms linking wildflower strips, wild bees, and honey bees are clearly warranted.

Trends of decreasing wild bee abundance and species richness relative to hive presence could also explain fruit count decreases. While previous research suggests the abundance of common species can have a greater impact on pollination services than species richness^53,54^, species richness could also enhance pollination efficiency in crops^55^. We observed enhanced winter squash fruit count with increasing species richness and not abundance, suggesting rarer species contributed crucial pollination services in winter squash. Our results also support past work suggesting pollination efficiency in strawberries is not associated with species richness^56,57^.

Our results documenting negative yield effects on farms with honey bee hives differ from several previous studies in agricultural systems. Two examples include a study in almond orchards in Northern California^15^, and another on sunflower production farms in Central California^13^ where honey bees enhanced outcrossing and increased yields. One difference between our study and those which found a beneficial effect is the scale of crop production involved. Because honey bees tend to seek out and concentrate in large floral resource patches^58^, they may pollinate crops in large acreage production more effectively than our small plantings of strawberries and winter squash. If the presence of honey bee hives decreased wild bee abundance on our study farms and honey bees were not visiting the strawberry or winter squash blooms, that could explain why we saw deficits in fruit count rather than a neutral or positive effect of honey bees. In fact, a recent study found that strawberries adjacent to mass-flowering oilseed rape received fewer honey and bumble bee visitations while the oilseed rape was blooming^59^. However, in contrast with our results, Bänsch and others found that farms benefitted from wildflower strips during mass oilseed rape blooms because the wildflower strips supported wild pollinators which stepped in to pollinate strawberries, replacing managed bees and protecting strawberry yields^59^.

The relative effectiveness of wild vs. honey bees as strawberry and winter squash pollinators differs in the literature. Prior research in strawberries observed roughly equivalent^56^ or more efficient pollination services by honey bees relative to wild bees^56,60–62^. However, a recent study found pollination from wild bees generated larger strawberries relative to pollination from honey bees alone or from a combination of wild and honey bees^57^, and Horth and Campbell compared strawberry production with pollination from managed honey bees with *Osmia lignaria*, an endemic species to the Mid-Atlantic, U.S., and found *O. lignaria* increased berry size and growth rates^63^. Relative to strawberries, cucurbits require a heavier pollen load to achieve maximum fruit count, and larger species such as bumble bees tend to be more efficient pollinators than honey bees^64,65^. Regardless, our observations of greater fruit count on farms without honey bee hives suggests wild pollinators populations in the southeastern Mid-Atlantic region of the USA may be robust enough to fulfill pollination requirements to an equal or greater degree than managed honey bees on small- or medium-scale farms. However, this should be verified in commercial crop fields to determine whether wild pollinator communities could support larger scale production needs. Two studies in the northeastern USA demonstrated that wild pollinators provide sufficient pollination services to pumpkins^66,67^, suggesting it is not outside the realm of possibility.

Whereas the decrease in fruit count relative to honey bee hive presence was clear, fruit count did not vary by honey bee density. This could indicate that the threshold of honey bees needed to affect wild bee communities and their services is quite low. Indeed, four out of the 11 farms which maintained hives at some point during our study kept them for only one season or year, but the effects on fruit count and wild bee abundance were nevertheless strong enough to detect. In a recent review, Geslin and others^68^ recommended limiting honey bees to ≤ 3.5 hives km^−2^ (0.035 hives ha^−1^) to conserve wild bees in protected areas^69,70^. For comparison, current recommendations range from 0.2 to 4 hives ha^−1^ to support strawberry production, and 0.02–1.2 hives ha^−1^ to support pumpkin and winter squash production^71–73^. Growers interested in wild pollinator conservation should carefully consider whether to rent or maintain honey bee hives, especially if accompanying pollination service benefits and associated costs are in question.

## Conclusion

The results of our study suggest that under some circumstances, supplementing farms with honey bee hives could detract from fruit count in pollinator-dependent crops and decrease wild bee abundance and species richness. Moreover, although wildflower strips provided a limited boost to fruit count, they did not alleviate honey bee hive-associated decreases in fruit count or decreases in wild bees on farms. As such, our study implies honey bee hives may detract from the goals of wildflower strips to enhance fruit count and support wild pollinator populations. Our research also suggests wild pollinators in the Mid-Atlantic region may have the potential to meet or exceed pollination services by honey bees in pollinator-dependent crops such as strawberry and winter squash. We suggest future work to investigate whether similar effects occur in larger-scale crop production, in other pollinator-dependent crop systems, and with regional variations in wild pollinator communities. More work identifying appropriate honey bee density thresholds relative to wild pollinator populations would benefit growers making management decisions and help them avoid financial costs or even yield reductions resulting from unnecessary hive rental.

## Materials and methods

We conducted research on farms (n = 21) in the Eastern Shore and Virginia Beach areas within the Mid-Atlantic USA (Fig. 1; Supplemental Table S1). Farms were small- or medium-scale (< 105 ha) with varied management practices and crops in production, and all were ≥ 2 km apart. All but three farms grew one or more pollinator-dependent crops, in which animal pollination is required to maximize production (Supplemental Table S1). We established wildflower strips on nine farms in 2016^74^, and one was established following the same guidelines in 2015. Mixes contained at least nine species of wildflowers (Ernst Conservation Seeds, Meadowville, PA; Roundstone Native Seed Company, Upton, KY), adapted for soil drainage conditions and to provide continual blooms spring through fall (Supplemental Table S2). Wildflower strip size varied according to grower preference and land availability (0.056–12.14 ha; μ = 0.22 ha ± 0.10 SE), but met or exceeded a size threshold at which detectable changes in wild bee density and/or diversity could be expected^75^. Approximately half of the farms maintained or rented honey bee hives (2017 early spring, WF + hives n = 5, WF − hives n = 5, control + hives n = 4, control − hives n = 7; 2017 mid-summer: WF + hives n = 7, WF − hives n = 3, control + hives n = 4, control − hives n = 7; 2018 early spring: WF + hives n = 4, WF − hives n = 6, control + hives n = 5, control − hives n = 5; 2018 mid-summer: WF + hives n = 4, WF − hives n = 6, control + hives n = 4, control − hives n = 6); the number and distance of hives to wildflower strips varied by farm and season (Supplemental Table S3). We surveyed floral resources within wildflower strips (n = 10 farms) and field margins (n = 10 farms) by counting blooms m^−2^ in early spring (May 7) and mid-summer (July 2) 2018 within a 1-m^2^ quadrat placed every 10 m along a 50-m transect. Blooming plants were identified to genus and species when possible.

### Bees

We surveyed the bee (Hymenoptera: Apidae) communities on farms in 2017 (n = 19) and 2018 (n = 20) during strawberry and winter squash bloom periods (Supplemental Table S3): two surveys were completed on each farm per year, occurring the weeks of May 14th and August 14th in 2017, and June 4th and August 13th in 2018. We placed nine bee bowls and three Blue Vane traps (Springstar Inc., Woodinville, WA, USA) out on each farm for 48 h. Bee bowls were made from 750 mL plastic food storage bowls (Rubbermaid, Atlanta, GA), with an equal number painted UV-bright yellow, blue, or white (Blick Art Materials, Galesburg, IL, USA). We included blue vane traps in the survey as an attempt to mitigate some of the disadvantages of pan traps, because they are effective at collecting larger bodied bees as well as bees which fly at higher elevations^41^. All bowls and traps were filled halfway with water and a drop of unscented dish soap. We placed them ≥ 2 m apart in a row along unmanaged field edges at control farms or wildflower strip edges at farms with plantings. Bowls were set on the ground in alternating colors, and blue vane traps were taped to three-foot posts at the center and both ends of the array. Collected bees were identified to species.

### Fruit count

To examine pollination services, we quantified total fruit count of strawberries (*Fragaria* × *ananassa* var. Chandler) and winter squash (*Cucurbita maxima* var. Gold Nugget) (Johnny’s Selected Seeds, Fairfield, ME, USA) in 2017 and 2018. Cultivated strawberry pollination occurs through a mix of self-, wind-, and insect-facilitation, but it is well established that increases in insect-facilitated pollination benefit yield^63,76^. Winter squash fruit development is entirely dependent on insect pollination^77^. Thus, fruit count is affected by insect pollination efficiency in both strawberries^78,79^ and winter squash^80^.

In order to minimize the effects of environmental variation across farms, we planted strawberries and winter squash in containers. We used two, 50-gal tubs (Rubbermaid, Atlanta, GA, USA) per farm augmented with drainage holes and filled with soil (Sun Gro, BFG, Burton, OH, USA). Containers were placed within field margins located 5–10 m from wildflower strips on farms which had them established. We added 25 g 5–10–10 fertilizer (Wetsel Fertilizer, BFG, Burton, OH, USA) upon transplanting strawberries or winter squash to each container, and again to strawberries ca. two weeks after transplanting. If transplants died, were smaller than average, or looked sickly, we replaced them with plants of the same size and growth stage within the first few weeks prior to the flowering stage. Two sites experiencing squash plant mortality during the flowering stage were excluded from the study in 2017. Four first-year strawberry plants were transplanted into each container in early April. Large chicken wire cages enclosed each bin of strawberry plants to exclude vertebrates. Ripened berries (≥ 75% pink or red surface) were collected weekly for seven weeks each year until plants stopped setting fruit in mid-June. We transplanted two five-week-old winter squash seedlings into each bin following strawberry plant removal in mid- to late-June. Winter squash fruits were collected when the connecting vine senesced and until plants either died or bore no more fruit in late September–early October. We removed and destroyed squash bugs (*Anasa tristis*) and eggs by hand from winter squash plants; if we observed larger outbreaks of squash bugs or cucumber beetles (*Acalymma vittatum*, *Diabrotica undecimpunctata*) and plants were not flowering, they were sprayed with Azera (MGK, Minneapolis, MN). Plants were watered on a weekly basis when container soil was dry to the touch.

### Data analyses

The number of farms under wildflower and honey bee hive presence/absence treatments involved in each analysis is presented in Supplemental Table S4. All post hoc comparisons of hive treatment means within each wildflower strip treatment were generated from full models containing interaction terms, but all nonsignificant interaction terms were subsequently dropped in the final analyses to more accurately gauge the significance of main effects. All models were constructed with R Studio v. 1.0.136^81^. Initially, the floral resource quantity and composition within field margins and wildflower strips in 2018 were compared. We calculated bloom density as the mean bloom counts m^−2^ and Shannon Wiener diversity indices [∑[(*p*_*i*_)x*ln*(*p*_*i*_), where *p*_*i*_ = the proportion of total specimens represented by species *i*] and compared them by season using Wilcoxon signed rank tests for mean blooms m^−2^ and a one-way ANOVA for Shannon Wiener diversity indices. A datapoint from one farm (HR AREC) in early spring was excluded from analyses because its value for bloom count m^−2^ was more than 50 times greater than the median.

#### Bees

To assess whether wildflower strips, honey bee hive presence, or their interaction on farms induced changes in conservation services, we first quantified wild bee abundance as the total number of non-*Apis* spp. bees per farm from all bee bowls and blue vane traps combined per survey date. We calculated bee species richness (S), Shannon–Wiener diversity (H), and species evenness per farm [H/ln(S)]. We omitted a datapoint from analyses driven by a spike in the abundance of a single species [*Agapostemon virescens* (Fabricius)] at one farm (M) from the June 2018 survey which generated outliers, making total wild bee abundance on the farm roughly ten times greater than the median for early spring in 2018. We then analyzed data with models containing hive treatment (presence/absence), wildflower strip treatment (presence/absence), and year fixed effects and their interactions, season (late May/early June vs. August) as a covariate, and farm as a random effect. We fitted wild bee abundance with a negative binomial distribution and species richness with a Poisson distribution using generalized linear mixed models (GLMMs), and analyzed diversity and evenness using linear mixed effects models (LMMs). We fitted models with the same distributions to examine 2018 wild bee abundance, species richness, diversity, and evenness data relative to the floral resources within field margins (control farms) or wildflower strips. Models contained bloom density and bloom species diversity m^−2^ per farm nested within the wildflower strip presence/absence treatment, a season covariate, two-way interactions with season, and a farm random effect. The GLMMs were analyzed using the glmmTMB package^82^ and LMMs with the lme4 package^83^. When year-by-treatment interaction terms were significant, we also ran pairwise post hoc tests on treatment effects by year with the emmeans package^84^. Additionally, we validated anecdotal evidence that feral honey bee hives in the region are rare and confirm honey bee abundance on farms differed by hive presence/absence. To do so, we analyzed total honey bee counts from bowls and traps per farm by hive treatment, year, and season fixed effects with farm as a random effect using a GLMM fitted with a negative binomial distribution.

#### Fruit count

To determine whether wildflower strips, honey bee hive presence, or their interaction enhanced fruit count on farms, we first calculated fruit count by year as the total number of strawberry or winter squash fruit produced per farm. We then analyzed total fruit count per farm by crop in fully factorial GLMMs, fitting strawberries with a negative binomial distribution to account for overdispersion and winter squash with a Poisson distribution. Predictor variables included year, hive treatment, wildflower strip treatment, and all two-way interactions with farm as a random effect. When significant treatment-by-year interactions occurred, we ran post hoc comparisons of treatment effects by year. Due to the limited number of farms producing winter squash during 2018, possibly due to heat suppressing female flower production^85^, replication was insufficient to include a wildflower strip-by-hive treatment interaction in the GLMM. Thus, we ran an additional GLM for 2017 winter squash fruit count data including a wildflower strip-by-hive interaction effect. We investigated fruit count relative to honey bee density by analyzing fruit count with separate GLMMs containing honey bee abundance, hive distance, or number of hives as predictor variables each fully crossed with year, and farm as a random effect.

To determine whether variation among wild bee populations corresponded to changes in fruit count, we regressed strawberry fruit count by bee data collected during the early spring and winter squash fruit count by the bee data collected during the summer sampling efforts. Separate GLMMs analyzed fruit count by wild bee abundance, diversity, species richness, or evenness predictor variables, each of which was fully crossed with year, with farm as a random effect. Again, we used a negative binomial distribution to analyze strawberry and a Poisson distribution to analyze winter squash fruit count.

## Supplementary Information

Supplementary Tables.

## Data Availability

The datasets generated during and/or analyzed during the current study were deposited in the Ag Data Commons, https://data.nal.usda.gov/dataset/data-honey-bee-hives-decrease-wild-bee-abundance-species-richness-and-fruit-count-farms-regardless-wildflower-strips.
