# Author Correction: Honey bee hives decrease wild bee abundance, species richness, and fruit count on farms regardless of wildflower strips

**DOI:** 10.1038/s41598-021-95368-x

**Published:** 2021-08-17

**Authors:** G. M. Angelella, C. T. McCullough, M. E. O’Rourke

**Affiliations:** 1 School of Plant and Environmental Sciences, Virginia Tech, Blacksburg, VA USA; 2 Present Address: USDA, Agricultural Research Service, Temperate Tree Fruit and Vegetable Research Unit, 5230 Konnowac Pass Road, Wapato, WA 98951 USA; 3 Present Address: Department of Entomology, Virginia Tech, Blacksburg, VA USA; 4 Present Address: USDA, National Institute of Food and Agriculture, Kansas City, MO USA

Correction to: *Scientific Reports* 10.1038/s41598-021-81967-1, published online 05 February 2021

The original version of this Article contained calculation errors in the dataset (wild bee abundance, species richness, diversity, and/or evenness miscalculations in five separate incidences). As a result of rerunning the 20 analyses reported in the manuscript involving this dataset, the statistical output changed to some extent in 15 of the analyses. The corrected dataset was resubmitted to the Ag Commons Data Library.

Consequently, in the Abstract,

“By contrast, wild bee abundance decreased by 48%, species richness by 20%, and strawberry fruit count by 18% across all farm with honey bee hives regardless of wildflower strip presence, and winter squash fruit count was consistently lower on farms with wildflower strips with hives as well.”

now reads:

“By contrast, wild bee abundance decreased by 49%, species richness by 22%, and strawberry fruit count by 18% across all farm with honey bee hives regardless of wildflower strip presence, and winter squash fruit count was consistently lower on farms with wildflower strips with hives as well.”

In the Results, under the subheading ‘Bees’,

“Wildflower strips did not significantly increase wild bee abundance [+ wf strips μ = 37.82 ± 5.73, − wf strips μ = 34.47 ± 5.82], species richness (+ wf strips µ = 8.36 ± 0.65, − wf strips µ = 7.42 ± 0.64), evenness (+ wf strips µ = 0.77 ± 0.033, − wf strips µ = 0.80 ± 0.026), or Shannon–Wiener diversity (+ wf strips µ = 1.53 ± 0.10, − wf strips µ = 1.42 ± 0.095) (Fig. 2A–D, Table 1). Wild bee diversity was significantly greater in mid-summer (µ = 1.67 ± 0.074) than early spring (µ = 1.29 ± 0.11) but did not differ by year (Table 1). Neither wild bee abundance, species richness, nor evenness differed by season or year (Table 1). Although wild bee species richness did not significantly differ by wildflower strip presence/absence on farms, it increased with wildflower strip bloom density (*Z* = 2.21, *P* = 0.027) but not bloom density in unmanaged field margins on control farms (*P* = 0.31) (Fig. 3B). However, bloom density did not affect wild bee abundance (field margins *P* = 0.63, wf strips *P* = 0.31), evenness (field margins *P* = 0.42, wf strips *P* = 0.94), or diversity (field margins *P* = 0.16, wf strips *P* = 0.21) (Fig. 3A,C,D), and bloom species diversity did not affect any wild bee metric: abundance (field margins *P* = 0.63, wf strips *P* = 0.31), species richness (field margins *P* = 0.60, wf strips *P* = 0.87), diversity (field margins *P* = 0.47, wf strips *P* = 0.87), or evenness (field margins *P* = 0.58, wf strips *P* = 0.57).

Honey bee hive presence was associated with a 48% decrease in wild bee abundance (+ hives μ = 24.00 ± 3.71, − hives μ = 46.31 ± 6.40), and a 20% decrease in species richness (+ hives μ = 6.94 ± 0.73, − hives μ = 8.69 ± 0.55) (Fig. 2A–B), whereas diversity (+ hives µ = 1.40 ± 0.12, − hives µ = 1.55 ± 0.077) and evenness (+ hives µ = 0.81 ± 0.034, − hives µ = 0.76 ± 0.026) did not differ significantly (Table 1).”

now reads:

“Wildflower strips did not significantly increase wild bee abundance [+ wf strips μ = 37.82 ± 5.73, − wf strips μ = 35.39 ± 5.73], species richness (+ wf strips μ = 8.38 ± 0.65, − wf strips μ = 7.66 ± 0.61), evenness (+ wf strips μ = 0.77 ± 0.033, − wf strips μ = 0.79 ± 0.024), or Shannon–Wiener diversity (+ wf strips μ = 1.62 ± 0.090, − wf strips μ = 1.55 ± 0.069) (Fig. 2A–D, Table 1). Wild bee diversity was significantly greater in mid-summer (μ = 1.68 ± 0.073) than early spring (μ = 1.47 ± 0.086) but did not differ by year (Table 1). Neither wild bee abundance, species richness, nor evenness differed by season or year (Table 1). Although wild bee species richness did not significantly differ by wildflower strip presence/absence on farms, it increased with wildflower strip bloom density (*Z* = 2.20 *P* = 0.028) but not bloom density in unmanaged field margins on control farms (*P* = 0.29) (Fig. 3B). However, bloom density did not affect wild bee abundance (field margins *P* = 0.63, wf strips *P* = 0.31), evenness (field margins *P* = 0.42, wf strips *P* = 0.94), or diversity (field margins *P* = 0.15, wf strips *P* = 0.21) (Fig. 3A,C,D), and bloom species diversity did not affect any wild bee metric: abundance (field margins *P* = 0.63, wf strips *P* = 0.31), species richness (field margins *P* = 0.55, wf strips *P* = 0.85), diversity (field margins *P* = 0.45, wf strips *P* = 0.88) , or evenness (field margins *P* = 0.57, wf strips *P* = 0.57).

Honey bee hive presence was associated with a 49% decrease in wild bee abundance (+ hives μ = 24.00 ± 3.71, − hives μ = 47.14 ± 6.30), and a 22% decrease in species richness (+ hives μ = 6.94 ± 0.73, − hives μ = 8.93 ± 0.51) (Fig. 2A–B), whereas diversity (+ hives μ = 1.58 ± 0.10, − hives μ = 1.59 ± 0.067) and evenness (+ hives μ = 0.81 ± 0.034, − hives μ = 0.76 ± 0.025) did not differ significantly (Table 1).”

In Table 1, the data in “WF”, “Hive”, “Year”, “Hive*Year” and “Season” columns were modified accordingly. The original Table 1 and accompanying legend appear below.

Further, under the subheading ‘Fruit count’,

“Winter squash fruit count increased with greater wild bee species richness (*Z* = 2.40, *P* = 0.017), but strawberry fruit count did not (*P* = 0.54) (Fig. 5A,B). Fruit count was unaffected by wild bee abundance (*P* = 0.52, *P* = 0.27), diversity (*P* = 0.91, *P* = 0.29), or evenness (*P* = 0.13, *P* = 0.23) in both strawberry and winter squash, respectively.”

now reads:

“Winter squash fruit count increased with greater wild bee species richness (*Z* = 2.52, *P* = 0.012), but strawberry fruit count did not (*P* = 0.51) (Fig. 5A,B). Fruit count was unaffected by wild bee abundance (*P* = 0.53, *P* = 0.27), diversity (*P* = 0.99, *P* = 0.25), or evenness (*P* = 0.14, *P* = 0.24) in both strawberry and winter squash, respectively.”

In Figure 5, the P and Z values were incorrect in panels A and B. The original Figure 5 and accompanying legend appear below.

In the Discussion,

“Meanwhile, there were marked overall decreases in wild bee abundance (48%), species richness (20%), and strawberry fruit count (18%) on farms with hives, regardless of wildflower strip presence or absence.”

now reads:

“Meanwhile, there were marked overall decreases in wild bee abundance (49%), species richness (22%), and strawberry fruit count (18%) on farms with hives, regardless of wildflower strip presence or absence.”

Additionally, upon reanalysis with the corrected dataset, one result changed from non-significant to significant: the post hoc comparison between wild bee abundance on wildflower strip farms in 2017 (corrected: *t* = 2.06 ± 0.37, *P* = 0.043; previous: *t* = 1.83 ± 0.44, *P* = 0.073). As a result, in Figure 2A, the pair ‘Wildflower strip’ was incorrectly indicated as non-significant. The original Figure 2 and accompanying legend appear below.

The original Article has been corrected.

**Figure 2:**
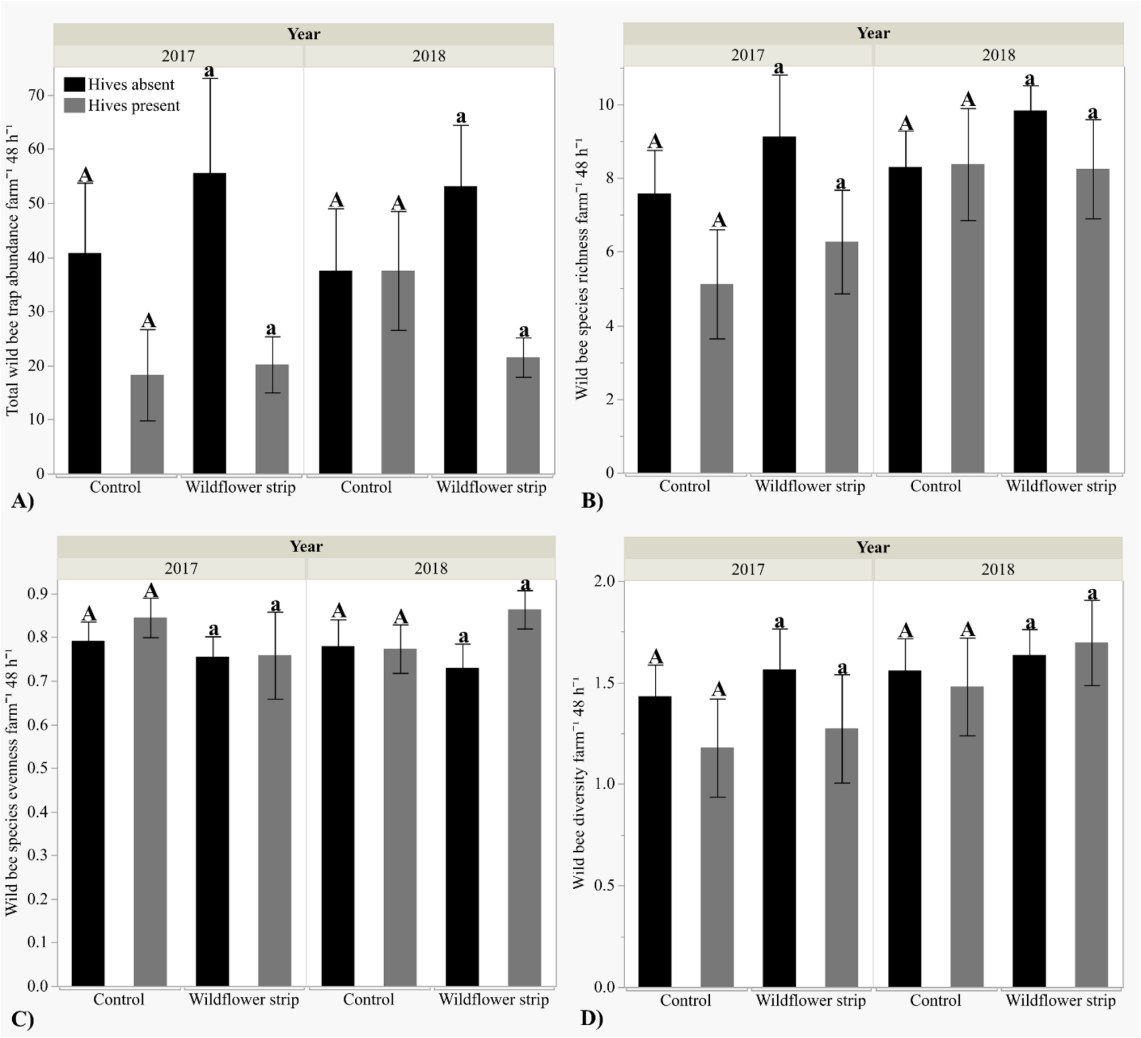
Mean total wild bee abundance, species richness, Shannon–Wiener diversity index, and species evenness (± SE) per farm by wildflower strip and honey bee hive presence/absence. Hive treatment means are compared within each wildflower strip treatment: the same letter indicates means are not statistically significant (α = 0.05). (**A**) Abundance, (**B**) Species richness, (**C**) Diversity, (**D**) Evenness.

**Table 1:** Wild bee survey data (abundance, species richness, Shannon–Wiener diversity, and species evenness) and fruit count data in response to wildflower strip (WF) and hive presence/absence, year, and/or season.

Response	WF	Hive	Year	WF*Year	Hive*Year	WF*Hive	WF*Hive*Year	Season
Wild bee abundance	*P* = 0.42	***Z* = −2.41 ** ***P* = 0.016**	*P* = 0.86	–^α^	*P* = 0.087	–^α^	–^α^	*P* = 0.13
Wild bee richness	*P* = 0.50	***Z* = −2.03, ** ***P* = 0.042**	*P* = 0.50	–^α^	*P* = 0.098	–^α^	–^α^	***Z* = 2.45, ** ***P* = 0.014**
Wild bee diversity	*P* = 0.42	*P* = 0.45	*P* = 0.085	–^α^	–^α^	–^α^	–^α^	***Z* = 3.10, ** ***P* = 0.0031**
Wild bee evenness	*P* = 0.81	*P* = 0.65	*P* = 0.88	–^α^	–^α^	–^α^	–^α^	***Z* = 2.08, ** ***P* = 0.043**
Strawberry fruit count	***Z* = 2.10, ** ***P* = 0.036**	***Z* = 2.43, ** ***P* = 0.0015**	***Z* = 5.77, ** ***P *< 0.001**	***Z* = −2.02, ** ***P* = 0.043**	–^α^	–^α^	–^α^	
Winter squash fruit count	*P* = 0.60	*P* = 0.34	***Z* = -3.15, ** ***P* = 0.0017**	–^α^	***Z* = −2.60, ** ***P* = 0.0093**	*– *^*β*^	*– *^*β*^	

Significant predictors (P < 0.05) are bolded.

^α^Nonsignificant interactions (α = 0.1) dropped from analyses.

^β^Interaction not included in analysis due to limited winter squash fruit count in 2018.

**Figure 5:**
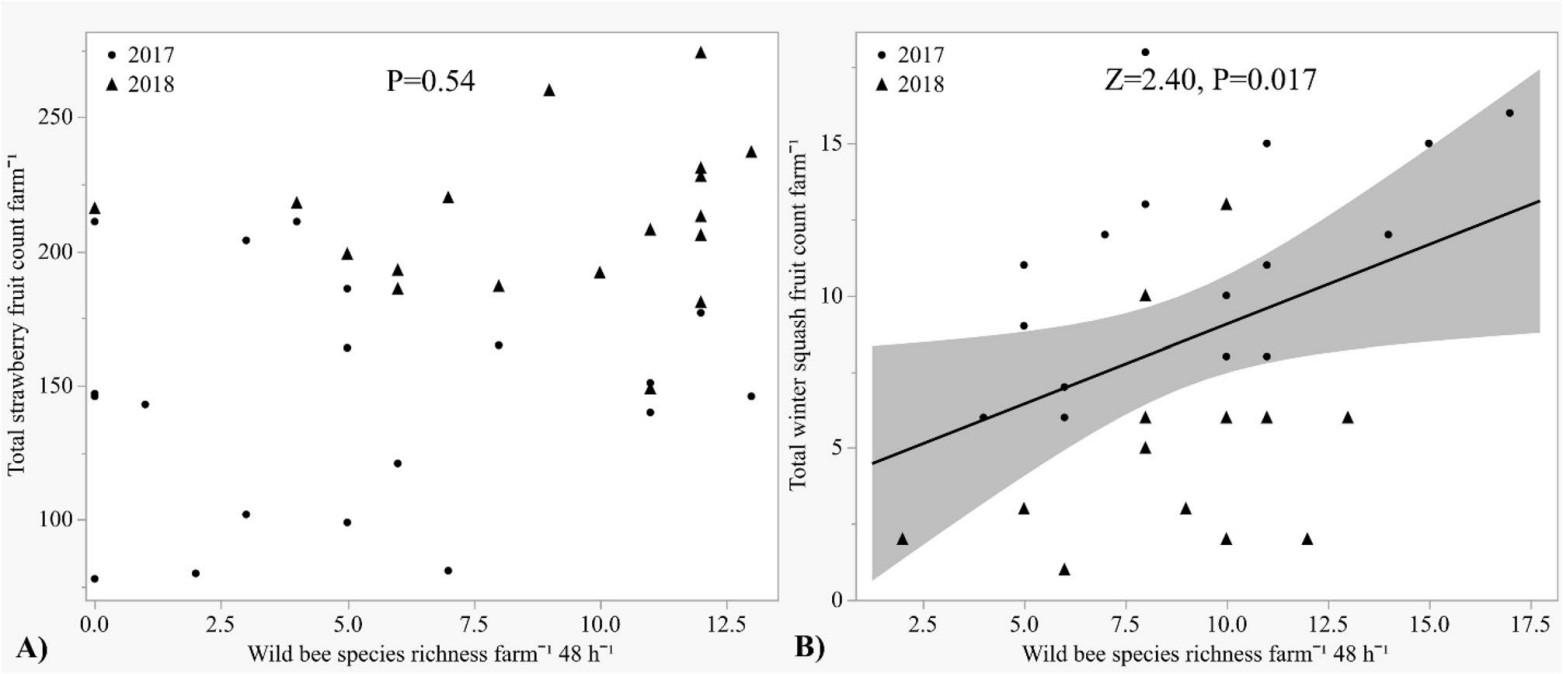
Total strawberry and winter squash fruit count analyzed by wild bee species richness: ▲ = 2017, ● = 2018. (**A**) Strawberry fruit count, (**B**) Winter squash fruit count.

