# Supplementary material for: Honey bee hives decrease wild bee abundance, species richness, and fruit count on farms regardless of wildflower strips
Source: Sci Rep. 2021 Feb 5;11:3202. doi: 10.1038/s41598-021-81967-1 (PMC7865060; doi:10.1038/s41598-021-81967-1)
Supplement: Supplementary file 1 — Supplementary Tables. [file 41598_2021_81967_MOESM1_ESM.docx]

Honey bee hives decrease wild bee abundance, species richness, and fruit count on farms regardless of wildflower strips

Angelella, G.M._1_^1*,2^

McCullough, C.T.^1,3^

O’Rourke, M.E.^1,4^

^1^School of Plant and Environmental Sciences, Virginia Tech, Blacksburg, VA, USA

^*^Corresponding author

^2^Present Address: USDA, Agricultural Research Service, Temperate Tree Fruit and Vegetable Research Unit, 5230 Konnowac Pass Road, Wapato, WA, 98951, USA, Gina.Angelella@usda.gov

^3^Present Address: Department of Entomology, Virginia Tech, Blacksburg, VA, USA, ctmccull@vt.edu

^4^Present Address: USDA, National Institute of Food and Agriculture, Kansas City, MO, USA, Megan.ORourke@usda.gov

**Supplemental Table S1.** Study farm locations, decimal degree coordinates, sizes, refuge treatments, and crops grown. Pollinator-dependent crops bolded.

| Farm | Treatment | Size (ha) | Latitude | Longitude | County | State | Crops |
| --- | --- | --- | --- | --- | --- | --- | --- |
| CA | pollinator refuge | 0.61 | 38.331° | -75.679° | Wicomico | MD | **Blackberries**, **cut flowers**, **lespedeza**, **pumpkins**, **squash** |
| CC | pollinator refuge | 0.81 | 37.393° | -75.949° | Northampton | VA | Arugula, basil, **blueberries**, cabbage, carrots, chard, celery, collards, **cut flowers**, fennel, figs, green beans, kale, lettuce, mulberries, onions, peppers, potatoes, snap beans, **squash**, **tomatoes**, wheat |
| CU | control | 101.17 | 36.626° | -76.035° | Virginia Beach | VA | **Apples**, beans, cotton, eggplant, peas, **pumpkins**, sweet corn, **squash**, **sunflowers**, **tomatoes** |
| ES AREC | pollinator refuge | 91.16 | 37.584° | -75.823° | Accomack | VA | Basil, barley, broccoli, cotton, eggplants, lettuce, peppers, potatoes, **pumpkins**, **rape seed**, rye, snap beans, soybeans, sweet corn, sweet potatoes, Swiss chard, **tomatoes**, vetch, **watermelons**, wheat |
| FLA | pollinator refuge | 6.88 | 36.716° | -76.015° | Virginia Beach | VA | Cabbage, eggplants, **pumpkins**, soybeans, **strawberries**, sweet corn, **tomatoes** |
| FLI | pollinator refuge | 8.09 | 36.706° | -75.992° | Virginia Beach | VA | **Blackberries**, broccoli, cabbage, cauliflower, green beans, peas, potatoes, **strawberries**, sweet corn, **tomatoes** |
| HR AREC | control | 28.33 | 36.893° | -76.179° | Virginia Beach | VA | **Ornamental plants**, **strawberries** |
| LC | control | 0.20 | 37.777° | -75.643° | Accomack | VA | Beans, **squash**, **tomatoes** |
| LESREC | control | 86.60 | 38.376° | -75.656° | Wicomico | MD | **Cantaloupes**, field corn, **grapes**, lima beans, **pumpkins**, soybeans, **sunflowers**, sweet corn, **watermelons** |
| M | control | 8.90 | 37.379° | -75.982° | Northampton | VA | Amaranth, brassicas, kale, potatoes, **tomatoes**, sweet corn |
| PG | control | 6.07 | 38.414° | -75.802° | Wicomico | MD | Asparagus, blackberries, **currants**, kale, mustard greens, onions, string beans, **squash**, **tomatoes**, turnips |
| PH | control | 76.89 | 38.357° | -75.777° | Wicomico | MD | Field corn, millet, soybeans |
| PP | pollinator refuge | 18.21 | 37.648° | -75.678° | Accomack | VA | Asparagus, beets, brassicas, fennel, **squash**, turnips |
| PO | control | 0.45 | 38.307° | -75.888° | Wicomico | MD | Beans, lettuce, peppers, **squash**, **tomatoes** |
| PR | pollinator refuge | 19.83 | 37.712° | -75.670° | Accomack | VA | Kale, lettuce, **pumpkins**, **squash**, Swiss chard, **tomatoes** |
| QC | control | 32.38 | 37.399° | -75.886° | Northampton | VA | **Squash**, **sunflowers**, sweet potatoes |
| SE | control | 22.26 | 37.431° | -75.870° | Northampton | VA | Cotton, soybeans |
| ST | pollinator refuge | 52.61 | 37.335° | -75.997° | Northampton | VA | **Pumpkins**, soybeans, **sunflowers**, wheat |
| UMES | pollinator refuge | 18.21 | 38.214° | -75.670° | Somerset | MD | Amaranth, **cucurbits**, **tomatoes** |
| VD | control | 40.47 | 37.811° | -75.630° | Accomack | VA | Carrots, horseradish, field corn, soybeans |
| W | pollinator refuge | 16.19 | 38.454° | -75.701° | Wicomico | MD | Beans, **squash**, **sunflowers**, sweet corn, **tomatoes**, **watermelon** |

**Supplemental Table S2.** Pollinator refuge wildflower species included in pollinator refuge mixes.

| Mix^ϯ^ | Species | Common name |
| --- | --- | --- |
| Well-draining soil, Poorly-draining soil, ST | *Chamaecrista fasciculata* (Michx.) Greene | Partridge pea |
| Well-draining soil, Poorly-draining soil, ST | *Coreopsis tinctoria* Nutt. | Plains coreopsis |
| Well-draining soil, ST | *Gaillardia pulchella* Foug. | Indian blanket |
| Well-draining soil, ST | *Rudbeckia hirta* L. | Blackeyed Susan |
| Well-draining soil, ST | *Coreopsis lanceolata* L. | Lanceleaf coreopsis |
| Well-draining soil | *Echinacea purpurea* (L.) Moench | Purple coneflower |
| Well-draining soil, ST | *Helianthus maximiliani* Schrad. | Maximilian sunflower |
| Well-draining soil | *Monarda fistulosa* L. | Wild bergamot |
| Well-draining soil | *Pycnanthemum tenuifolium* Schrad. | Narrowleaf mountain mint |
| ST | *Monarda punctata* L. | Spotted bee balm |
| ST | *Oenothera speciosa* Nutt. | Showy evening primrose |
| Well-draining soil, Poorly-draining soil | *Bidens aristosa* (Michaux) Britton | Showy tickseed |
| Well-draining soil, Poorly-draining soil | *Solidago nemoralis* Ait. | Gray goldenrod |
| Poorly-draining soil | *Symphyotrichum puniceum* var. puniceum | Purple-stemmed aster |
| Poorly-draining soil | *Helenium autumnale* L. | Common sneezeweed |
| Poorly-draining soil | *Solidago rugosa* Mill. | Wrinkleleaf goldenrod |
| Poorly-draining soil | *Eupatoriadelphus fistulosus* (Barrett) | Spotted Joe pye weed |
| Poorly-draining soil | *Eryngium yuccifolium* Michaux. | Rattlesnake master |
| Poorly-draining soil | *Hibiscus moscheutos* L. | Rosemallow |
| Poorly-draining soil | *Helianthus angustifolius* L. | Narrowleaf sunflower |

**Supplemental Table 3.** Honey bee hive locations, numbers, and distance to sentinel crop plants by year and early (Apr–Jun) or late (Jul–Sep) seasonal timing. Early or late seasonal sentinel crops (strawberry, winter squash) grown, bee surveys, and bee reproduction data measured on each farm indicated as well.

|  |  | April–June | | | | July–September | | | |
| --- | --- | --- | --- | --- | --- | --- | --- | --- | --- |
| Farm | Year | Strawberry | Bee survey | Hives (n=) | Distance to hives (m) | Winter Squash | Bee survey | Hives (n=) | Distance to hives (m) |
| CA^PR^ | 2017 | X | X | 1 | 25 | X |  | 1 | 25 |
|  | 2018 | X | X | 0 | -- | X | X | 0 | -- |
| CC^PR^ | 2017 | X | X | 0 | -- | X | X | 0 | -- |
|  | 2018 | X | X | 0 | -- | X | X | 0 | -- |
| CU | 2017 | X | X | 3 | 100 | X | X | 8 | 3x100, 5x400 |
|  | 2018 | X | X | 3 | 100 | X | X | 3 | 100 |
| ES AREC^PR^ | 2017 | X | X | 0 | -- | X | X | 0 | -- |
|  | 2018 | X | X | 0 | -- | X | X | 0 | -- |
| FLA^PR^ | 2017 | X | X | 4 | 300 | X | X | 4 | 300 |
|  | 2018 | X | X | 0 | -- | X | X | 0 | -- |
| FLI^PR^ | 2017 | X | X | 5 | 175 | X | X | 5 | 175 |
|  | 2018 | X | X | 5 | 175 | X | X | 5 | 175 |
| HR AREC | 2017 | X | X | 3 | 500 | X | X | 3 | 500 |
|  | 2018 | X | X | 2 | 500 | X | X | 3 | 500 |
| LC | 2017 | X | X | 0 | -- | X | X | 0 | -- |
|  | 2018 | X | X | 0 | -- | X | X | 0 | -- |
| LESREC | 2017 | X | X | 20 | 91 | X | X | 20 | 91 |
|  | 2018 | X | X | 10 | 91 |  |  | 10 | 91 |
| M | 2017 | X | X | 0 | -- | X | X | 0 | -- |
|  | 2018 | X | X^*^ | 0 | -- | X | X | 0 | -- |
| PG | 2017 | X | X | 0 | -- | X | X | 0 | -- |
|  | 2018 | X | X | 0 | -- | X | X | 0 | -- |
| PH | 2017 |  |  | 0 | -- | X | X | 0 | -- |
|  | 2018 | X | X | 0 | -- | X | X | 0 | -- |
| PP^PR^ | 2017 | X | X | 0 | -- | X | X | 8 | 4x300, 4x500 |
|  | 2018 | X | X | 0 | -- | X | X | 0 | -- |
| PO | 2017 | X | X | 0 | -- | X | X | 0 | -- |
|  | 2018 |  |  | 0 | -- |  |  | 0 | -- |
| PR^PR^ | 2017 | X | X | 2 | 25 |  | X | 2 | 25 |
|  | 2018 | X | X | 2 | 25 | X | X | 2 | 25 |
| QC | 2017 | X | X | 0 | -- | X | X | 0 | -- |
|  | 2018 | X | X | 40 | 425 | X | X | 0 | -- |
| SE | 2017 |  |  | 0 | -- | X | X | 0 | -- |
|  | 2018 | X | X | 0 | -- | X | X | 0 | -- |
| ST^PR^ | 2017 | X | X | 0 | -- | X | X | 8 | 300 |
|  | 2018 | X | X | 0 | -- | X | X | 0 | -- |
| UMES^PR^ | 2017 | X | X | 0 | -- | X | X | 0 | -- |
|  | 2018 |  | X | 1 | 400 |  | X | 1 | 400 |
| VD | 2017 | X | X | 1 | 27 | X | X | 1 | 27 |
|  | 2018 | X | X | 1 | 27 |  | X | 1 | 27 |
| W^PR^ | 2017 | X | X | 2 | 500 | X | X | 2 | 500 |
|  | 2018 | X | X | 2 | 500 | X | X | 6 | 2x500, 4x250 |

*Removed from analyses due to outlier.

Supplemental Table 4. Wildflower strip (WF) and honey bee hive presence/absence treatments (n=number of farms) by season and year. Farm counts reflect only those included in analyses. A) Bee abundance, B) Fruit count.

A)

| Year | Season | WF+hives | WF-hives | Control+hives | Control-hives |
| --- | --- | --- | --- | --- | --- |
| 2017 | Early spring | 5 | 5 | 4 | 5 |
| 2017 | Mid-summer | 6 | 3 | 4 | 7 |
| 2018 | Early spring | 4 | 6 | 5 | 5 |
| 2018 | Mid-summer | 4 | 6 | 3 | 6 |

B)

| Year | Season | Crop plant | WF+hives | WF-hives | Control+hives | Control-hives |
| --- | --- | --- | --- | --- | --- | --- |
| 2017 | Early spring | Strawberries | 5 | 5 | 4 | 5 |
|  | Mid-summer | Winter squash | 7 | 3 | 4 | 7 |
| 2018 | Early spring | Strawberries | 3 | 6 | 5 | 5 |
|  | Mid-summer | Winter squash | 3 | 5 | 2 | 6 |
